# Synthesis and Toxicity of Graphene Oxide Nanoparticles: A Literature Review of *In Vitro* and *In Vivo* Studies

**DOI:** 10.1155/2021/5518999

**Published:** 2021-06-10

**Authors:** Asmaa Rhazouani, Halima Gamrani, Mounir El Achaby, Khalid Aziz, Lhoucine Gebrati, Md Sahab Uddin, Faissal AZIZ

**Affiliations:** ^1^Laboratory of Water, Biodiversity & Climate Change, Cadi Ayyad University, B.P. 2390, 40000 Marrakech, Morocco; ^2^National Centre for Studies and Research on Water and Energy (CNEREE), Cadi Ayyad University, B. P 511, 40000 Marrakech, Morocco; ^3^Laboratory of Clinical, Experimental and Environmental Neurosciences, Cadi Ayyad University, Marrakech, Morocco; ^4^Materials Science and Nano-Engineering (MSN) Department, Mohammed VI Polytechnic University (UM6P), 43150, Benguerir, Morocco; ^5^Materials, Catalysis and Valorization of Natural Resources, Faculty of Sciences, University Ibn Zohr, BP 8106 Agadir, Morocco; ^6^Laboratory of Materials, Processes, Environment and Quality, Cadi Ayyad University, BP 63, 46000 Safi, Morocco; ^7^Department of Pharmacy, Southeast University, Dhaka, Bangladesh; ^8^Pharmakon Neuroscience Research Network, Dhaka, Bangladesh

## Abstract

Nanomaterials have been widely used in many fields in the last decades, including electronics, biomedicine, cosmetics, food processing, buildings, and aeronautics. The application of these nanomaterials in the medical field could improve diagnosis, treatment, and prevention techniques. Graphene oxide (GO), an oxidized derivative of graphene, is currently used in biotechnology and medicine for cancer treatment, drug delivery, and cellular imaging. Also, GO is characterized by various physicochemical properties, including nanoscale size, high surface area, and electrical charge. However, the toxic effect of GO on living cells and organs is a limiting factor that limits its use in the medical field. Recently, numerous studies have evaluated the biocompatibility and toxicity of GO *in vivo* and *in vitro*. In general, the severity of this nanomaterial's toxic effects varies according to the administration route, the dose to be administered, the method of GO synthesis, and its physicochemical properties. This review brings together studies on the method of synthesis and structure of GO, characterization techniques, and physicochemical properties. Also, we rely on the toxicity of GO in cellular models and biological systems. Moreover, we mention the general mechanism of its toxicity.

## 1. Introduction

Nanoparticles are widely used in electronics, aeronautics, energy, agriculture, cosmetics, medicine, textile production, and many other fields. They are currently used to administer drugs, proteins, genes, vaccines, polypeptides, and nucleic acids [1]. According to the International Organization for Standardization, a nanomaterial is defined as a material with at least one external dimension at the nanoscale. That is to say between approximately 1 and 100 nm or that has an internal or surface structure at the nanoscale [2]. Apart from their nanoscale size, nanoparticles can be classified according to their shape or chemical composition. Depending on their chemical composition, carbon-based nanomaterials exist in nature in many different forms. They are used in science and technology for drug delivery [3], cell imaging [4], and cancer therapy [5]. GO is a nanomaterial that has been known for more than 150 years [6] and is used in many applications. It is the precursor of graphene, an excellent two-dimensional material that is part of the carbon allotropes. It was discovered in 2004 by Andre Geim's team at the University of Manchester in England [7]. Graphene is characterized by the diversity of its physicochemical properties, including thermal property [8], electrical conductivity [9], mechanical strength [10], and transparency [11]. Grace to these properties, graphene is used in many fields, such as water desalination [12], electronics [13], and desorption/ionization [14]. In recent years, graphene has been exploited in the medical field, particularly for DNA sequencing [15], the development of biosensors, and cell differentiation and growth [16]. As graphene is insoluble in water, its applications are limited to passive platforms for detection and cell work. Its functional derivative GO has unique properties that make it more effective for biomedical applications. It is characterized by its ability to disperse in many solvents, facilitating its handling [17]. In addition, GO is used to administer anticancer drugs in biological cells [18], aptamers for ATP probing in mouse epithelial cells, and gene delivery [19]. These nanomaterials have a large surface area and can maintain drugs' stability without altering the biological activity, much more than other nanomaterials [20]. Previous studies have shown that multifunctional dressings produced from biomechanically active self-healing injectable hydrogels based on quaternized chitosan (QCS), polydopamine-coated reduced graphene oxide (rGO-PDA), and poly(N-isopropylacrylamide) (PNIPAm) to promote wound closure and healing. These dressings adhere strongly to the skin and promote wound closure by actively contracting wounds through self-contraction [21]. Other works have prepared a series of antibacterial, adhesive, hemostatic, antioxidant, conductive, photothermal and hyaluronic acid, dopamine, and reduced graphene oxide- (rGO-) based hydrogels using H2O2/HPR system that can improve the complete regeneration of the skin. This makes it very interesting for clinical applications [22]. In addition, it has been shown that supramolecular hydrogel-based dressings produced from polymer solutions of quaternized chitosan-graft-cyclodextrin (QCS-CD), quaternized chitosan-graft-adamantane (QCS-AD), and graphene oxide-graft-cyclodextrin (GO-CD) have a conductivity value similar to that of the skin and a rapid self-healing behavior and have a high antibacterial property against bacteria [23]. Furthermore, another study developed a series of injectable antimicrobial conductive hydrogels based on quaternized chitosan (QCSG) functionalized with glycidyl methacrylate, gelatin methacrylate (GM), and graphene oxide for infectious wound healing and disinfection of drug-resistant bacteria. The results of this study showed that these hydrogels have good effects on the repair of infectious skin tissue [24]. GO is characterized by properties that make it attractive in other areas such as sensors [25] and energy storage [26]. As applications increase, exposure to GO increases across populations. These include exposures during nanomaterial manufacturing and biomedical treatment. GO is involved in many applications, but there is one main factor limiting “its toxicity” limiting its use. Researchers are often faced with the problem of balancing the positive therapeutic effects of GO with the side effects associated with its toxicity. For this reason, the choice of an experimental model, either *in vivo or in vitro*, must be of paramount importance in testing the toxicity of this nanoparticle. The toxic effects of GO depend on several factors, including the route of administration, the dose to be administered, the method of synthesis of GO, and its physicochemical properties. These factors influence and increase the complexity of comparisons between different studies on the toxicity of GO.

In this review, we have introduced the synthesis methods, structure, characterization techniques, and GO properties. In addition, we presented and discussed available toxicological studies of GO in vitro and in vivo. We summarized the cytotoxicity of GO in cellular models. We then focus on the pathways by which GO enters the body and the role of biological barriers. We also present the biodistribution, biotransformation, and excretion of this nanomaterial and also discuss the toxicity of GO in different body systems. Finally, we showed the general mechanism of toxicity, to better understand the toxic effects related to the exposure of GO to improve the biological safety of this nanomaterial and facilitate its use in the biomedical field.

## 2. Synthesis of GO

### 2.1. Brodie-Staudenmaier-Hummers Based Methods

The first synthesis of GO is often attributed to Brodie. In 1859, British researcher Benjamin Brodie carried out research that mainly consisted of oxidizing graphite sheets using potassium chloride (KClO_3_) is fuming nitric acid [27]. Brodie determined by elementary analysis that the product obtained was composed of carbon, oxygen, and hydrogen. He gave the term “graphic acid” to refer to his material.

Almost 40 years after Brodie's discovery, the German chemist Staudenmaier has reproduced Brodie's method, modifying specific parameters. This method consists of slowly mixing potassium chloride with a solution of sulfuric acid, concentrated nitric acid, and graphite. The mixture is kept under agitation for one week in a cooled environment. This modification increased the oxidation rate of the graphite sheets [28].

**Figure 1:**
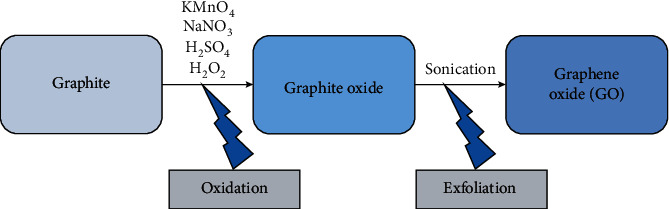
Diagram of GO preparation.

Almost a hundred years after Brodie's discovery, the chemists' Hummers and Offeman [29] published a new process for synthesizing graphite oxide, thereby reducing the risk of explosion and reaction time. They used a mixture of sulfuric acid, sodium nitrate, and potassium permanganate at a temperature of around 45°C for 2 hours to obtain a brownish-grey pasty [29]. The suspension was diluted with water, and hydrogen peroxide (H_2_O_2_) was added to get a higher oxidation degree and to eliminate manganese from the dispersion (Figure 1). Any method that modifies or improves the route of synthesis proposed by Hummers is considered a “Modified Hummers.” The synthesis route of GO can be changed according to the needs of each researcher. In general, the size and shape of the carbon source will determine the GO [30]. The average diameter of the graphite powders used in the synthesis will evaluate the average lateral dimension of the GO.

### 2.2. Tour Method

The Tour group proposed improving the Hummers method at the University of Rice in 2010 [31]. They have substituted the sodium nitrate with phosphoric acid in a mixture of H_2_SO_4_/H_3_PO_4_ (9 : 1) and increased KMnO_4_. This method's advantage is the absence of generation of toxic gases, such as NO_2_, N_2_O_4_, or ClO_2_, in the reaction, easy temperature control, and gives GO powders a higher degree of oxidation.

### 2.3. Free-Water Oxidation Method

In 2013, Sun and Fugetsu of Hokkaido University [32] introduced a more direct method for producing GO. They used expanded graphite as a carbon precursor. Potassium permanganate had a double effect: intercalating agent and oxidizing agent. The intercalation of KMnO_4_ between the graphitic layers produces another spontaneous expansion that resembles foam of graphitic material. The reaction takes place in the middle of sulfuric acid.

Two years later, Peng and his collaborators [33] proposed a way of producing GO, using potassium ferrate (K_2_FeO_4_) as a strong oxidant. In this method, a mixture of graphite powder and K_2_FeO_4_ dispersed in concentrated sulfuric acid was loaded into a reactor and stirred for 1 hour at room temperature. The product was washed with water by repeated centrifugation to obtain highly water-soluble GO.

Pendolino and his collaborators have improved another procedure called the 4-step method [34]. It consists of 4 reaction steps controlled by temperature, which strongly affects the final product. The first step consists of mixing the graphite with KMnO_4_ and in the presence of concentrated sulfuric acid, resulting in the formation of pasty slurry. The second step requires the exfoliation of the graphite. Indeed, the production of GO is limited by temperature and only occurs when the water bath is at about 30°C. Hydrolysis at 90°C for 1 hour completes the third step. Purification of the product is carried out by centrifugation with hot water until the dispersion is neutral in the fourth step. This method's advantage is related to the improved operational safety conditions and the production of a type of GO that contains less than about 20-30% oxygen domains. This type of GO can be used for filtering/remediation or biosystems due to the common toxic effect.

In all of the above synthesis methods for preparing GO, certain limitations are encountered. The use of sodium nitrate or potassium chlorate in the Brodie or Staudenmaier methods leads to explosive results. In contrast, sodium nitrate (Hummers) or fuming nitric acid introduces heteroatoms or defects on the GO structure that affects reactivity [29]. Another critical factor is the quality and grain size of the graphite. Indeed, a defect-free structure gives better GO quality [35], and the grain size establishes the format for graphene's basal plane.

### 2.4. Monolithic Crystalline Swelling of GO

Recently, a more innovative study of the synthesis of the GO has been proposed. In this study, researchers prepared the ultrawide GO (medium size from 108 *μ*m and the largest size from 256 *μ*m) using a swelling crystal strategy using oxidation-monolithic crystal swelling that can ultimately convert graphite into the ultrawide GO [36]. This new strategy minimizes the reduction in GO sheet size and inhibits the onset of gelling so that the resulting graphite oxide can be purified quickly and easily. The oxidized graphite flakes undergo monolithic crystal swelling during purification, resulting in the formation of an ordered three-dimensional structure. On the other hand, this strategy is necessary to develop advanced devices and high-performance nanocomposites.

To sum up, there is no specific method or procedure for producing a “standard” GO because each synthesis method produces a different GO type. Therefore, the GO has distinct physicochemical properties, such as structure and reactivity. The discrepancy between the structure and reactivity of the GO is in most cases due to the synthetic method and the carbon source, as reported in the literature. Standardization of the synthesis appears to be one of the main challenges to applying GO for advanced applications. Nevertheless, the production of different GO types by other synthesis methods can broaden this nanomaterial's implementation by modulating its properties and opening new promising opportunities for exploitation.

## 3. Structure of GO

**Figure 2:**
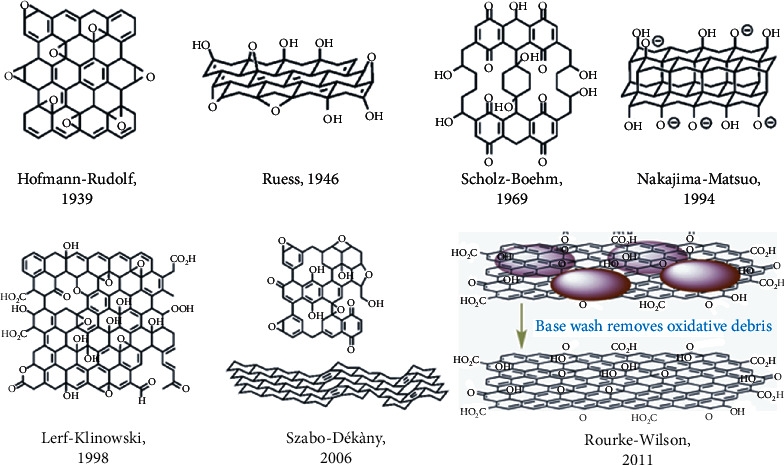
Different structures of GO.

Over the years, several structures have been suggested for the GO (Figure 2), starting with Hofmann's in 1939 [37]. Hofmann and Holst proposed a model in which epoxide groups distributed throughout the graphene plane give a C/O ratio of 2. In this model, the carbon skeleton is of sp^2^ hybridization. Subsequently, Ruess introduced hydroxyl groups into the model in 1947 [38]. He proposed that the carbon structure was composed of cyclohexane repeating units of sp^3^ hybridization. In 1969, Scholz and Boehm suggested a different model in which epoxide groups were removed from the GO structure and replaced by ketones [39]. In 1994, Nakajima and Matsuo proposed a remarkable model in which two layers of GO were linked together by covalent bonds of C-C type sp^3^ hybridization [40]. These C-C bonds were perpendicular to the surface of the GO bilayer. The model proposed by Nakajima and Matsuo replaced the epoxy and ether groups of the structure with ketones and hydroxyl groups sporadically distributed on the surface of GO. In 1998, Lerf and Klinowski [41] proposed a model with features such as a nearly flat carbon grid structure with aromatic regions randomly distributed with unoxidized benzene and areas with six-membered aliphatic rings. They also pointed out that the carbon atoms attached to the OH groups may slightly distort their tetrahedral structure, resulting in some folding of the layer. This model proposed by Lerf and Klinowski is the most recognized. Eight years later, Szabó and his colleagues modified the Scholz and Boehm model. They offered a carboxylic acid-free model with two distinct domains: translinked cyclohexyl species intercalated with tertiary alcohols and 1,3-ethers and a wavy network keto/quinoidal species [42]. Subsequently, in 2011, Rourke and his collaborators proposed a sophisticated model of washed GO based on oxidizing debris, showing a structure quite different from those previously suggested [43]. In 2013, other researchers proposed a new dynamic structural model. This model explained the acidity of aqueous GO solutions [44]. Recently, Liu et al. [45] directly observed the oxygen binding, and they proposed a structural model with C=O bonds on its edge and plane, which partly confirms the models proposed previously.

## 4. Characterization of GO

There are different analytical techniques used to characterize the physicochemical properties of GO. These include techniques such as atomic force microscopy (AFM), scanning electron microscopy (SEM), transmission electron microscopy (TEM), Raman spectroscopy, solid state nuclear magnetic resonance (Ss-NMR), Fourier transform infrared spectroscopy (FT-IR), X-ray induced photoelectron spectroscopy (XPS), X-ray diffraction (XRD), and thermogravimetric analysis (TGA).

### 4.1. Atomic Force Microscopy (AFM)

Atomic force microscopy is a technique used in nanotechnology to study materials at the nanoscale. It is capable of characterizing the lateral size and thickness of the GO layer. In general, the height profile reveals a diameter of 1–1.2 nm. Simultaneously, the lateral size can be in the order of tens to hundreds of micrometers, depending on the synthesis and postsynthesis processing, e.g., sonication. Analyses obtained by this technique have shown that the GO layers' thickness is about 1.1 nm and a lateral size ranging from 500 nm to 50 *μ*m [46].

**Figure 3:**
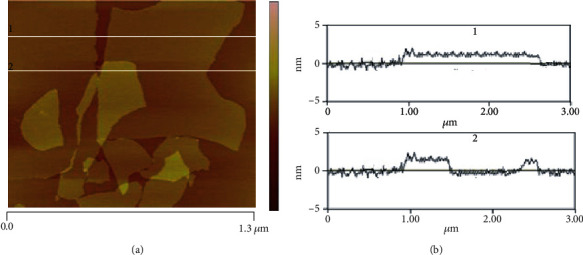
Atomic force microscopy image (a) and a monolayer's thickness profile of graphene oxide (b).

Atomic force microscopy profiling (Figure 3) shows GO particles with lateral dimensions of 1 to 1.5 *μ*m (Figure 3(a)) and thickness of 1.5 to 2.5 nm (Figure 3(b)).

### 4.2. SEM and TEM Electron Microscopy

SEM and TEM electron microscopy use a high-energy electron beam to examine the material at a very detailed level [47]. The SEM technique is based on the principle of electronic scanning, and it provides all available information on nanoparticles at the nanoscale. The SEM results showed that the GO's morphology appears as a tight layer with a wavy surface that is sometimes wrinkled [34]. Similarly, TEM is based on the electron transmission principle to provide information on the material's dimensions. The analyses obtained by TEM are useful for identifying a single layer of GO. This technique has shown the GO layer's wavy or pleated structure that is highly transparent to electrons. Typical examples of unique and multilayer GO layers are presented by Aunkor [48] and Wang [49].

**Figure 4:**
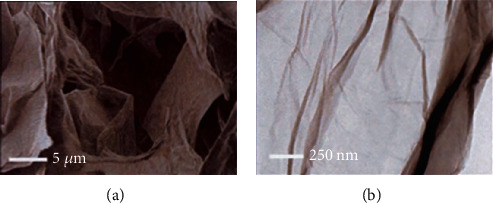
SEM (a) and TEM (b) electron microscopy images of GO monolayers.

The scanning electron microscopy image (Figure 4(a)) shows large and intact OG sheets entangled on top of each other in the material after lyophilization. At higher magnification, the transmission electron microscopy image shows that the OG monolayers are almost transparent and are structurally free (Figure 4(b)).

### 4.3. Raman Spectroscopy

Raman spectroscopy is based on the phenomenon of inelastic scattering of a monochromatic light beam [50]. This technique mainly consists of observing the vibrational and rotational modes in a material. This spectroscopy type allows the acquisition of information on molecular vibrations to identify several compounds' chemical structures. The Raman spectrum for GO shows only two broadened peaks that represent the G and D bands. The first band is at about 1580 cm^−1^ and is attributed to the ordered crystal structure's phase vibrations, while the D band (∼1350 cm^−1^) is attributed to the disorder's crystal structure. This fact correlates G-band to carbon sp^2^ and D-band to the presence of sp^3^ and thus to oxygen domains [45, 46].

**Figure 5:**
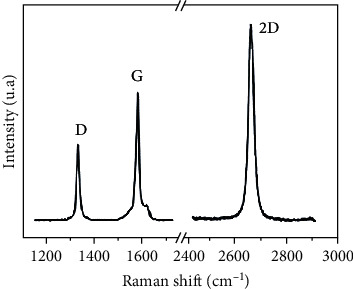
Raman spectrum of the graphene sheet.

The Raman analysis was presented in Figure 5. The spectrum shows four vibration bands which are characteristic of sp^2^ hybridized carbon materials. The vibration band G (around 1580 cm^−1^) represents the atoms' vibrations in the plane and corresponds to a permanently active mode.

The D band (around 1335 cm^−1^) corresponds to a vibration mode called breathing, which does not obey the selection rules that determine whether a mode is active.

The 2D band (around 2700 cm^−1^) is a harmonic of the D-band and provides information on the graphene planes' stacking order. In monolayer graphene, the 2D band is an intense Lorentzian peak (about four times the intensity of band G). The shape of the 2D strip then changes according to the number of planes, then from five planes of thickness, becomes identical to that of hexagonal graphite.

### 4.4. Solid-State Nuclear Magnetic Resonance (Ss-NMR)

**Figure 6:**
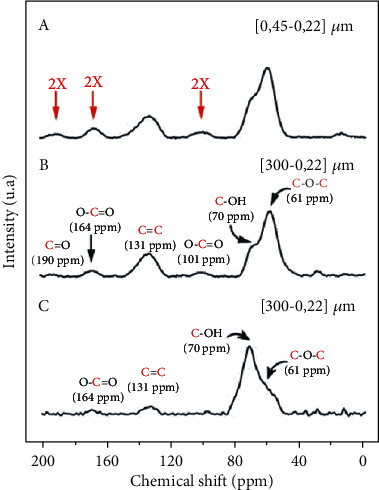
Pulsed carbon-13 (^1^H-decoupled) NMR spectra of GO with particle sizes between 0.45 *μ*m and 0.22 *μ*m (a), from GO to unfractionated particles (b) and ^1^H →13C cross-polarized NMR spectrum, and from GO to unfractionated particles (c).

The use of pulsed solid-state NMR on carbon-13 (PD) with rotation at the magic angle (12.5 kHz) makes it possible to identify the GO structure's different carbon atoms characteristic.  Figure 6 shows that the integration of the NMR signals of an OG sample with small particles with dimensions between 0.45 and 0.22 *μ*m (Figure 6(a)) is twice as important for the oxygen functions at the periphery (red) compared to an unfractionated LO sample (Figure 6(b)). Small OG slips have a periphery/surface ratio greater than the large sheets. Consequently, the intensity of the NMR signals attributed to the functions C = O carbonyl (190 ppm), 0-C = O carboxylic acid (164 ppm), and 0-C-0 hydroxy-lactone (101 ppm) found on the border is higher for small particles because they have more border per unit area.

### 4.5. Fourier Transform Infrared Spectroscopy (FT-IR)

Fourier transform infrared spectroscopy is a technique for studying chemical bonds' movement by measuring the absorption of electromagnetic radiation from a compound. This analysis is based on the excitation of molecular bonds in a sample by infrared radiation (2.5 to 50 *μ*m) of frequencies between 4000 and 200 cm^−1^ [51]. It is a useful tool for the rapid characterization of GO. The FT-IR signals are interpreted as hydroxyl (OH), epoxide (C-O-C), and ketone (C=O) signals. The results obtained by the FT-IR confirmed the existence of oxygen-containing groups on the GO nanosheets in which the main absorption band at 3340 cm^−1^ is attributed to the stretching vibrations of the O-H group. The absorption peak at 1730 cm^−1^ and 1630 cm^−1^ can be attributed to the C=O stretching of the carboxyl and carbonyl parts' functional groups. The two absorption peaks at about 1226 cm^−1^ and 1044 cm^−1^ are attributed to the C-O group's stretching vibrations [52].

**Figure 7:**
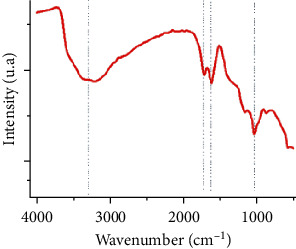
GO FTIR specter.

The result of the graphene oxide FTIR analysis is shown in Figure 7. The most significant transmittance bands in the spectra include the stretching and in-plane deformation of O-H bonds in the hydroxyl groups found, respectively, at 3380 cm^−1^ and 1365–1145 cm^−1^, the C=O carbonyl stretching at 1720 cm^−1^, the phenol C=C ring stretching at 1622 cm^−1^, and the epoxide group C > O vibration at 979 and 1041 cm^−1^. The band at 3645 cm^−1^ is assigned to H_2_O molecule, indicating that this molecule is intercalated into GO.

### 4.6. X-Ray Induced Photoelectron Spectroscopy (XPS)

X-ray induced photoelectron spectroscopy is a technique that provides information on the electron structure, organization, and morphology of the surface of a material. The XPS analysis of the GO showed significant C and O signals corresponding to the binding energy of the GO [53]. These analyses showed that GO sheets contain many functional groups on their surface, such as C-O and C=O.

**Figure 8:**
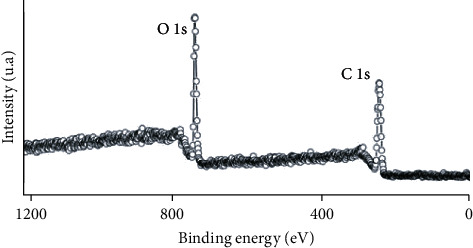
Scan range specter for the XPS analysis of GO.

The GO flyover XPS spectrum (Figure 8) shows the Ols and Cls band's characteristic signals around 532-533 eV and 285-286 eV, respectively. In XPS, the intensity of the signals, expressed in arbitrary units (a.u.), is proportional to the sample elements' abundance. Therefore, the relative intensity of the C1s and O1s bands makes it possible to directly calculate the carbon-oxygen (C/0) ratio of the compound, which is very useful in assessing the degree of oxidation of GO, for example.

### 4.7. X-Ray Diffraction (XRD)

**Figure 9:**
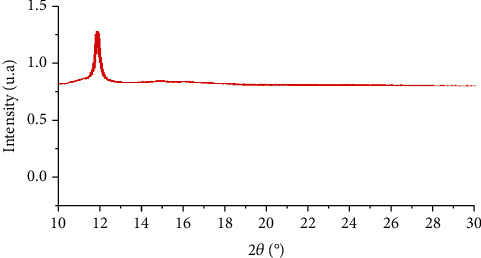
Specter range for the XRD analysis of GO.

X-ray diffraction is one of the most important characterization techniques for revealing nanoparticles' structural properties. It provides sufficient information about the crystallinity of nanoparticles. Analyses performed by X-ray diffraction have shown that phase analysis (Figure 9). That indicates the presence of three different carbon structures: hexagonal graphite (PDF file no. 04-007-2081), a set of turbostratic carbon, and orthorhombic carbon. The presence of hexagonal graphite is mainly indicated by the peak (002) located at 12°: the interplanar distance along the c⃗ axis is approximately equal to 340 pm, which is slightly higher than the interplanar distance recorded for hexagonal graphite (335 pm) and can be explained by the curvature of the graphene planes and the presence of defects. The set of turbostratic carbon denotes several graphitic structures with a lattice parameter c more or less high (varying from 824 to 4325 pm), which is to say with a more or less important stacking order the c⃗ axis.

The XRD analysis of GO in other work showed that the interlayer distance is 1 nm due to functional groups' presence on the GO [54]. This distance varies according to the solvent in which the GO is dispersed. Other researchers have reported a minimum interlayer distance of 0.82 nm for ethanol, and 1.17 nm for GO is dispersed in dimethylformamide (DMF) [55].

### 4.8. Thermogravimetric Analysis (TGA)

Thermogravimetric analysis is a destructive analysis technique based on the pyrolysis of a sample at high heat to analyze its contents [56]. Carbon-based samples are typically heated to 30-1000°C in an inert or oxidizing atmosphere [31]. This technique consists of measuring the change in mass of a sample (%) as a function of time for a specific temperature or temperature gradient applied to the sample. Thermogravimetric analyses of the GO have shown that initial weight loss occurs at about 100°C due to water molecules' loss. Significant weight loss for the GO was observed around 200°C and 250°C due to functional groups' decomposition [57].

**Figure 10:**
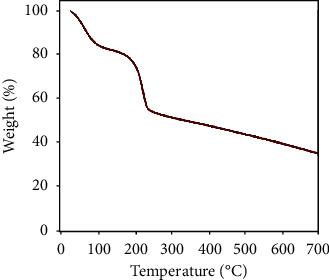
Thermogravimetric analyzes (TG-TGD) of GO sheets.

Figure 10 shows the results obtained by differential thermogravimetric analysis (TGD). The data on which the OG has three bearings indicate a significant loss of mass. The first level, around 106°C, represents the loss of mass associated with the water's evaporation (10%) in the OG. The second mass loss (45%) between 150 and 300°C reaches its maximum around 190-215°C. This plateau represents the loss of labile functions of the hydroxyl type on the surface of the OG. Finally, the third level, between 450 and 700°C, represents the loss of mass (35%) caused by the decomposition of the OG's more stable functions, such as the carboxylic acid groups and the phenols and also by the expulsion of carbon monoxide (CO) and carbon dioxide (CO_2_), during the pyrolysis of the carbon skeleton.

## 5. Properties of GO

GO is characterized by the diversity of its physicochemical properties. The main characteristic is that it behaves like a hydrophilic material, thanks to the hydroxyl groups, epoxides, ketones, and carboxylic acids. The presence of these oxygenated groups considerably modifies the properties of GO. They allow biochemical and bioconjugation reactions to occur at the basal plane and the edges of the GO [58]. These reactions facilitate this nanomaterial's surface's functionalization with proteins, antibodies, and DNA fragments [52, 53]. Also, GO shows a high specific surface area (890 m^2^g^−1^) [59] and mechanical resistance [60], and it is a semiconductor nanomaterial due to its degree of oxidation [61]. Studies have shown that conductive biomaterials are good candidates for use as scaffolds in muscle tissue engineering due to their excellent conductivity and influence on muscle tissue formation which allows GO to be more used in the medical field [62]. Particle size and specific surface area are essential parameters that play a significant role in the interaction of nanomaterials with the outside world. As a particle's size decreases, its specific surface area increases, and the number of atoms on the surface becomes more considerable [63]. This makes it possible to increase the ability of nanoparticles to penetrate the body's tissues. That is to say that these particles can cross-specific biological barriers in the body.

GO has a high adsorption capacity for proteins and antibodies. Proteins adsorbed in GO have been shown to increase protection against proteolysis [64]. The mechanism of interaction of proteins with the surface of GO varies according to its morphology, hydrophobicity, and the type of protein adsorbed [65]. On the other hand, it has been shown that the adsorption behavior of GO changes from Freundlich-type to Langmuir-type as the degree of oxidation increases [64]. Furthermore, concerning the protein type, the polypeptide can be adsorbed on the GO's surface by hydrophobic interaction, van der Waals forces, electrostatic interactions, and hydrogen bonds [59–62, 66]. Due to sp^2^ hybridization, protein adsorption on the GO's surface occurs by hydrophobic-hydrophobic interaction [67], causing the hydrophobic protein side to interact with the hydrophobic carbon network [68]. Besides, GO and other molecules' interaction can be attributed to van der Waals interactions [69]. Still, these interactions are weakened by the oxygen fractions formed during oxidation [70]. However, electrostatic interactions are most observable at GO sites [65]. On the other hand, hydrogen bonding interactions have shown how the adsorption of nitrogen oxides on GO is more reliable than on graphene due to OH-O (N) hydrogen bonds between -OH and nitrogen oxides, among others [71]. Finally, it should be mentioned that due to the abundance of electrons *π* on the basal plane of the GO surface, the stacking interactions *π*-*π* may also occur [72].

GO is characterized by the diversity of its physicochemical properties. It is hydrophilic, biocompatible, has a high specific surface, a mechanical resistance, and a semiconductor nanomaterial because of its oxidation degree. On the other hand, GO has a high adsorption capacity for proteins and antibodies, and it is considered superb catalyst support.

## 6. Toxicity of GO in Cell Models

The toxicity of GO in cells is due to several factors, including dose, lateral size, and surface charge [65, 73]. To date, the studies carried out on the cytotoxicity of GO are contradictory. Some studies have shown that GO has no toxic effects on cellular behavior, while others have reported that this nanomaterial can induce cellular damage. Studies have shown that GO can significantly promote cell growth by improving mammalian cell attachment and proliferation [74]. Other studies have indicated that GO can effectively enhance cell adhesion and proliferation with excellent biocompatibility. These positive interactions between GO and cells can be elucidated from the chemical structure of GO. It has been suggested that the wealthy oxygen-containing functional groups are responsible for their adequate support of cell adhesion and growth [75]. It has also been found that GO effectively provides vital signals and soluble factors for cell adhesion and growth [76].

On the contrary, there are several studies on the toxic effects of GO on cells. Researchers have reported that incubation of human breast cancer cells MDA-MB-231 with GO caused a decrease in cell viability due to the dose of GO exposure [77] (Table 1). Indeed, the cytotoxicity of this nanomaterial *in vitro* is closely related to incubation conditions, including exposure dose, culture time, incubation temperature, and cell type [78]. Also, the physicochemical properties of GO, such as shape, particle size, number of layers, and surface functionalization, affect the behavior of GO on cells. All these factors can bring about a variety of biological responses. For example, GO with a dose less than 20 *μ*g/mL did not exhibit toxicity to human fibroblast cells, and the dose of more than 50 *μ*g/mL exhibits cytotoxicity such as decreasing cell adhesion, inducing cell apoptosis, and entering into lysosomes, mitochondrion, endoplasm, and the cell nucleus [79].

Similarly, the dose of 20 *μ*g/mL showed no cytotoxicity on A549 cells [80]. The metabolic activity of neuronal PC12 cells decreased in a dose-dependent manner after one day of incubation with GO, affecting the mitochondrial activity and cell membrane integrity but still exhibiting cytotoxicity even at low concentrations [81]. The dependence of cytotoxicity on dose changes with different cell types. Indeed, the effect of GO on the human neuroblastoma cell line SH-SY5Y showed no cytotoxicity up to the 80 *μ*g/mL concentration of GO, observing a dose- and time-dependent reduction in viability at higher concentrations [82].

The lateral size of the GO also affects cytotoxicity. Researchers have shown that cytotoxicity depends on the lateral size and density of the functional groups of the GO. They found this result from exposure of human lung cells (BEAS-2B) and alveolar epithelial cells (A549) to three types of GOs that differ in lateral size and functional group density. They also found that GO and thermally reduced GO are more toxic than chemically reduced GO [83]. Another study by Chang and colleagues showed that GO with a smaller size caused more severe oxidative stress and induced more obvious cytotoxicity in A549 cells compared to GO with a larger size [80]. One study found that cell uptake of GO is size-dependent [84]. Researchers separated the GO sheets into different sizes and studied the nanomaterial size effect in response to different cell types. GOs of 2 *μ*m and 350 nm have very different lateral dimensions but also contribute to the amount of absorption in macrophages. Similar amounts of antibody opsonization and active Fc*γ* receptor-mediated phagocytosis have been shown to cause this behavior. While the microdimensional GO showed different intracellular locations and induced much stronger inflammatory responses.

Another critical factor inducing cytotoxicity is the surface charge of the GO. Studies have suggested that GO also has an impact on cell internalization and absorption [85]. The interaction between the GO and the cell membrane can cause morphological changes and cell lysis, such as hemolysis of red blood cells. These changes are due to strong electrostatic interactions between negatively charged oxygen groups on the GO's surface and positively charged phosphatidylcholine on the outer membrane of red blood cells [86]. On the other hand, the negative charge on the surface of the GO induced platelet activation and aggregation compared to the reduced GO functionalized with an amine (rGO-NH2). The latter could not produce a significant effect on the same doses [87].

Recently, a new study evaluated the toxicity of GO in the rat cardiomyoblast H9c2 cell line. It demonstrated that GO induced cardiotoxicity, mitochondrial disruption, generation of reactive oxygen species, and DNA interactions [88]. Based on the *in vitro* toxicity of GO in the literature, it can be said that this nanomaterial can be either harmless or toxic to cells. The degree of toxicity is a function of the physicochemical properties of GO and the experimental conditions.

## 7. Toxicity of GO In *Vivo*

### 7.1. Pathways of GO Entry into the Body and Biological Barriers

**Figure 11:**
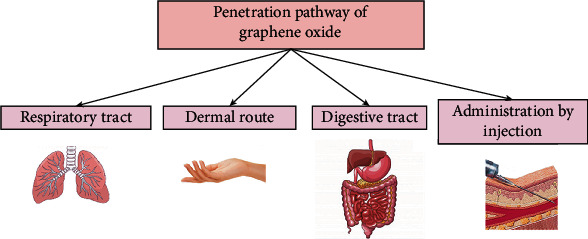
Pathways of GO entry into the body.

The natural routes of entry of nanoparticles into an organism are inhalation, ingestion, and dermal. As a result, some toxicological studies in animal models mimic this natural contamination pattern by directly bringing nanoparticles into contact with organisms. Others choose to administer GO by intravenous, intraperitoneal, and subcutaneous injections (Figure 11). These injections are also used for biomedical applications [89]. Studies have shown that intratracheal administration of GO in mice developed fibrosis in lung tissue after 21 days. Besides, in cells, GO increased the rate of mitochondrial respiration and the generation of reactive oxygen species, activating inflammatory and apoptotic pathways [90]. In addition to respiratory exposures, GO, after entering the body by intravenous injection, could also be retained in the lung and induce the formation of granulomas and pulmonary edema [91]. Also, inhaled GO nanosheets can destroy the ultrastructure and biophysical properties of pulmonary surfactant film, which is the host's first line of defense, and reveal their potential toxicity [92]. Once deposited at the bottom of the pulmonary alveoli, nanoparticles can be taken up by macrophages [93] or eliminated by respiratory mucus via the action of hair cells [94] or, for the smallest of them, pass through the pulmonary epithelium and end up in the interstitial liquid [95].

GO is considered to be an excellent drug delivery system [101]. It is usually incorporated with anticancer drugs to improve oral bioavailability [102]. Oral administration has shown that graphene nanosheets are mainly found in mice's stomachs and intestines [103]. Fu and colleagues evaluated the toxicological mechanism caused by GO. They found that the length of the filial mice's intestinal villus given a high concentration of GO orally was significantly reduced compared to the control group [104].

On the other hand, studies have suggested that intestinal absorption of nanoparticles is limited after oral administration, and their excretion is rapid [105]. Other studies have shown that after intraperitoneal injection, the GO mainly remained accumulated near the injection site. Simultaneously, small agglomerates could be found in the liver and spleen's serous membrane [103].

Moreover, GO-based nanomaterials have been structured as topical antimicrobial media in the form of bandages, ointments [106], or cotton fabric [89]. For this reason, dermal exposure is an important route of exposure that deserves attention. Xu and colleagues used Ag-reduced graphene oxide (Ag-rGO) on rat skin. They found that this exposure did not cause any skin irritation [107]. Furthermore, Zhao et al. [108] synthesized cotton GO-based antibacterial and reported no skin irritation in rabbits [108]. On the other hand, information on the dermal toxicity of GO is minimal, and much research is needed to understand the toxicological mechanisms better.

Other biological barriers are also mentioned in the literature. Studies have shown that GO particles with a 54.9 ± 23.1 nm diameter had difficulty penetrating the hemato-testicular and hemato-epididymal barriers after intra-abdominal injection. Also, the sperm quality of the mice was not affected, even at a dose of 300 mg/kg [109]. Regarding the placental barrier, one study suggested that the placenta does not provide a barrier against the transfer of nanoparticles to the fetus, specifically against the distribution of nanoparticles in and to the fetus [110]. Other studies on the blood-brain barrier have found that reduced graphene oxide (with a mean diameter of 342 ± 23.5 nm) is capable, over time, of inserting itself into the interendothelial cleft and decreasing the paracellular seal of the barrier [111].

### 7.2. Biodistribution, Biotransformation, and Excretion of GO

The biodistribution, biotransformation, and excretion of GO can be influenced by several factors, including routes of administration, physicochemical properties, particle agglomeration, and surface coating. Zhang et al. [91] found that GO is firmly retained in different organs such as the lungs, liver, spleen, and bone marrow after intravenous administration in mice. They also observed pulmonary edema in mice's lungs after intravenous injection of 10 mg/kg body weight of GO [91]. Similarly, GO polyethylene glycol (GO-PEG) functionalized derivatives are mainly retained in the reticuloendothelial system, including the liver and spleen, after intraperitoneal injection. However, these GO-PEG derivatives do not show any tissue absorption by oral administration [103]. However, the diameter of the GO influences its distribution (Table 2). Studies have shown that GO nanosheets with a diameter of 10-30 nm were found primarily in the liver and spleen, while nanosheets with a diameter of 10-800 nm were accumulated mainly in the lungs [52, 98, 99]. The coating of biocompatible polymers on GOs also affects biodistribution. For example, modifying the surface of GO, such as GO-PEG or GO-dextran (GO-DEX), facilitates the accumulation of this nanomaterial in the reticuloendothelial system without short-term toxicity [89, 100].

GO can undergo significant biotransformation and modify its physicochemical properties due to its greater chemical reactivity [112]. Qi and his colleagues showed that GO could undergo a significant physicochemical transformation in two simulated human lung fluids: Gamble's solution and artificial lysosomal fluid (ALF). Treatment of GO with these lung fluids reduced this nanomaterial, changing the carbonyl and epoxy groups into phenolic groups. This modification inhibited the endocytosis of GO by removing macrophages. Besides, the transformations occurring in Gamble's solution reduced the interaction of GO with cells and allowed its precipitation.

In contrast, ALF changes enhanced the adhesion of large sheet-like GO aggregates to the plasma membrane without cell uptake [113]. Other studies have shown that the biotransformation of GO in blood plasma influenced its toxicity. Free radicals and biological molecules in human blood plasma simultaneously caused a biological crown on biodegraded GO nanosheets. This biotransformation affected the interactions of the GO with cells. As well, the biotransformed GO induced lower levels of reactive oxygen species and damage to cell ultrastructure. Metabolomic analyses indicated that biotransformation reduced the oxidative stress induced by GO primarily by increasing fatty acid metabolism and decreasing galactose metabolism [114]. In some recent work, it has been shown that GO particles are aggregated by interaction with digestive fluids and the acidic pH of the stomach. However, no structural changes or degradation have been detected, indicating that GO is not biotransformed by oral absorption [115]. *In vivo* experiments in mice confirmed morphological alterations of the GO in a realistic lung microenvironment. These results suggested that the biotransformation of GO may significantly alter their inherent properties and thus affect their biosafety [113].

GO excretion varies in different organs. In the lungs, GO is challenging to eliminate, causing inflammation, cell infiltration, granuloma formation, and pulmonary edema [100, 105]. In the liver, GO nanoparticles can be eliminated through the hepatobiliary pathway by following the duodenum's bile duct [116]. Moreover, GO polyethylene glycol's functional derivatives accumulate primarily in the liver, and the spleen can be eliminated gradually, probably via the kidneys and fecal excretion. Besides, GO particles with large size of 200 nm are trapped by splenic physical filtration.

In contrast, small particles of about 8 nm can enter the renal tubules in the urine and be rapidly removed without any toxicity [117]. The routes of eliminating GO in vivo have not yet been clearly explained, but renal and fecal routes appear to be the major elimination routes. To date, several controversial results have been obtained regarding the distribution and excretion of this nanomaterial.

### 7.3. Toxicity in the Respiratory System

In order to examine the pulmonary toxicity of GO, studies have used a single 6-hour inhalation of GO at low and high concentrations in rats. After this exposure, the animals were allowed to recover for 1 day, 7 days, or 14 days. The results of this exposure showed that the levels of microalbumin and lactate dehydrogenase in the bronchoalveolar lavage (BAL) fluid were not significantly altered. Similarly, the total number of macrophages, leukocytes, and lymphocytes was not significantly altered in the BAL fluid. Moreover, histopathological analyses of rat lungs showed the only GO absorption in alveolar macrophages in the high concentration group [118]. Based on these results, it can be said that inhalation exposure to GO induced minimal toxic responses in the rats' lungs that received the high concentration.

**Figure 12:**
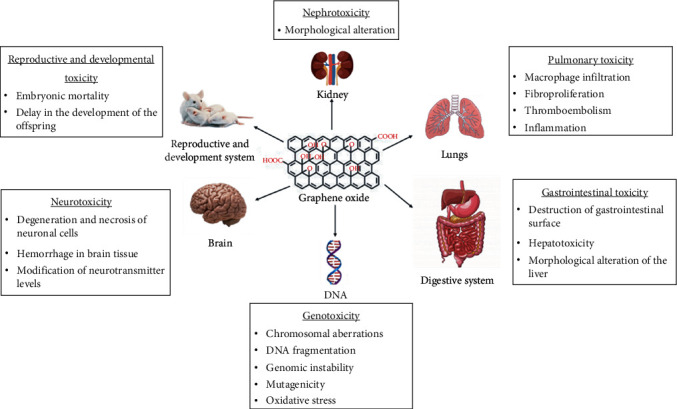
Effects of GO on organs.

On the other hand, intratracheal instillation of GO in vivo resulted in pulmonary toxicity. Li et al. [119] found that intratracheally instilled GO nanosheets can be retained in the lungs. This exposure resulted in acute lung injury and chronic pulmonary fibrosis. They found that these GO-induced acute lung lesions are related to oxidative stress. Also, histopathological examination revealed that GO induced fibroproliferation and organization of lung tissue in the acute phase. However, intravenous administration of GO caused massive pulmonary thromboembolism in mice (Figure 12). The prothrombotic character of GO was dependent on the distribution of the surface charge [120]. Direct administration of GO into the lungs of mice resulted in severe and chronic lung damage. These GO nanosheets disrupted the alveolar-capillary barrier, allowing inflammatory cells to infiltrate the lungs and stimulate the release of proinflammatory cytokines [90].

### 7.4. Toxicity in the Digestive System

Oral gavage experiments in animals found that GO was not absorbed from the gastrointestinal tract [121]. Fu and his colleagues found that low-dose GO caused severe damage to the gastrointestinal tract in maternal mice rather than high-dose GO. This is because the low dose of GO without agglomeration can easily attach to the gastrointestinal surface and cause destruction by its abundant sharp edges [104]. Furthermore, the study of the toxicity of GO in male rats who received different doses of this nanomaterial by the oral route showed hepatotoxic effects and induction of oxidative stress [122]. Consequently, this exposure caused an increase in liver enzymes' activity and morphological alteration of the liver tissue.

### 7.5. Toxicity in the Urinary System

Work has shown that GO is a nephrotoxic product and that its toxicity can be mediated by oxidative stress [123]. These studies found that administration of GO at different doses (10, 20, and 40 mg/kg) for five days significantly increased the activities of superoxide dismutase, catalase, and glutathione peroxidase in a dose-dependent manner in the kidney compared to the control group. Moreover, serum creatinine and blood urea nitrogen levels were also significantly increased in GO intoxicated rats than in the control group. Histological sections of the kidneys showed morphological alterations in GO intoxicated rats. In contrast, a new study demonstrated that intraperitoneal injection of GO in male albino mice did not cause kidney failure. This study's results did not show any significant change in urea and creatinine concentration in mice poisoned by GO [124].

Furthermore, histological analyses did not reveal any toxicity in the renal tissue. These results indicate that the injected GO nanoparticles do not have a toxic impact on the mice after 4 weeks of injection. In parallel, Jasim et al. observed significant urinary excretion after intravenous administration of GO to mice [125]. They observed no significant renal function changes or structural damage to the kidneys' glomerular and tubular regions up to one month after injecting the GO at increasing doses. Also, serum and urinalysis revealed no alterations in renal function. Also, histological examination revealed no lesions of the glomerular and tubular regions of the kidneys. From these studies, it can be said that the toxicity of GO on the kidneys has shown contradictory results, so that several studies are needed to understand this phenomenon better.

### 7.6. Toxicity in the Central Nervous System

GO and graphene-based nanomaterials have been widely used in recent years in biomedical applications to treat brain tumors, intracranial and spinal biocompatible devices, and biosensing and bioimaging techniques. However, the potential health risk and neurotoxic potential of GO are not yet clear. Amrollahi et al. [126] evaluated the in vivo toxicity of GO in Wistar rats. The results of their study showed that GO has a toxic effect on nerve tissue. Indeed, microscopic sections' analyses revealed that the cerebral and cerebellar cortex's specific neuronal cells showed degeneration and necrosis. In particular, the shape of Purkinje cells was disrupted, their cytoplasm was narrowed, and their nuclei disappeared. These changes were most noticeable in animals that received a high dose of GO. Besides, bleeding in brain tissue was observed in animals with GO intoxication. Also, no morphological changes were observed in the meninges and white matter. Another recent study has shown that GO can produce neurotoxic effects in the nematode *Caenorhabditis elegans* [127]. Indeed, exposure to GO caused a significant decrease in neurotransmitters such as tyrosine, tryptophan, dopamine, tyramine, and GABA. In addition, the decrease in fluorescence of Pgcy-8: GFP, which is a marker of sensory neurons, suggested that GO is capable of causing damage to these neurons. Besides, exposure to GO caused a decrease in the expression of ttx-1 and ceh-14 genes, which are genes necessary for sensory neurons' functioning. A significant change in locomotor behavior markers, such as speed, acceleration, and stopping time, was observed. These results provided information on the neurotoxic potential of neurotransmitters and sensory neurons in the nematode *Caenorhabditis elegans*.

On the other hand, Rauti and his colleagues [128] have proposed that small nanosheets of GO reduce glutamate availability, which is the major excitatory neurotransmitter in the central nervous system. The reduction of this neurotransmitter occurs by promoting its rapid release and subsequent depletion, leading to a decline in glutamatergic neurotransmission. Besides, they injected s-GO into the hippocampus in vivo, and 48 hours postoperatively, ex vivo patch-clamp recordings of brain slices show a significant reduction in glutamatergic synaptic activity compared to saline injections. However, another study showed that after intravenous administration of GO at different doses (2.5, 5, or 10 mg/kg of GO) for seven days and behavioral assessment in rats, GO did not affect the locomotor activity and exploratory behavior. Histopathological analyses also demonstrated that rats treated with GO did not undergo any cerebral cortex changes [129]. The toxicity of GO to the central nervous system requires further study to understand better how neurotoxicity occurs.

### 7.7. Toxicity in Reproductive and Development System

Studies have shown that GO and rGO are capable of causing damage to zebrafish embryos. Exposure to different concentrations of these nanomaterials influenced the hatching rate and body length of embryos. However, no malformations or mortalities were observed in zebrafish embryos after exposure to these two nanomaterials [130]. Another study revealed that the GO was adhered and wrapped in zebrafish embryos' chorion, causing hypoxia and delayed hatching. In addition, GO aggregates were retained in different regions, such as the eyes, heart, yolk sac, and tail of embryos. In these organs, GO induced apoptosis and excessive generation of reactive oxygen species and increased oxidative stress and DNA damage [131]. In parallel, a recent study has shown that GO is capable of inducing cardiovascular defects in zebrafish during development. However, the presence of GO at a low concentration (0.1-0.3 mg/mL) does not affect embryonic development, whereas the presence of GO at higher concentrations (0.4-1 mg/mL) induces significant embryonic mortality, increased heart rate, delayed hatching, cardiovascular defects, increased apoptosis, and decreased hemoglobinization [132].

Further work has shown that male mice given high doses of GO (25 mg/kg mice) by intravenous injection exhibited normal sex hormone secretion and maintained regular reproductive activity. All untreated females mated with male mice intoxicated with GO were able to produce healthy offspring. Histological analyses of the testes and epididymis with the activities of several epididymal enzymes, including *α*-glucosidase, lactate dehydrogenase, glutathione peroxidase, and acid phosphatase, were not affected by GO treatment [109]. On the other hand, Fu and his colleagues studied the toxic effects of GO on the development of offspring mice during the lactation period. This study showed that the increase in body weight, body length, and tail length of the filiform mice who received the GO during the lactation period was significantly delayed compared to the control group. Analysis of the histological sections revealed a delay in the offspring's development in the high-dose group of the GO. Also, they found that the length of intestinal villus of the filial mice that received a high concentration of GO was significantly reduced compared to the control group [104].

### 7.8. Genotoxicity

Studies have shown that GO is capable of inducing genotoxicity. Liu and his colleagues found that GO induced mutagenesis at the molecular level. The use of GO at concentrations of 10 and 100 *μ*g/mL altered gene expression. Furthermore, they showed that intravenous injection of GO at 4 mg/kg for 5 days in mice induced the formation of micronucleated polychromatic erythrocytes [77]. Another study was performed to investigate the genotoxic potential of different doses of GO in mice. The results of this study indicated that GO caused chromosomal aberrations in bone marrow cells and DNA fragmentation in lung cells as a function of time and injection dose [133].

On the other hand, another study showed that GO did not induce significant genotoxicity in FE1 murine pulmonary epithelial cells even at relatively high doses (5-200 *μ*g/mL) [134]. Therefore, a recent study demonstrated that after injection of GO at different doses (10, 20, and 40 mg/kg) for one or five consecutive days, it caused genomic instability, mutagenicity, and oxidative stress in the liver and brain tissue. Besides, administration of GO significantly increased dose-dependent DNA breaks and induced mutations in the p53 (6 and 7) and presenilin (exon 5) genes by increasing the expression of the p53 protein [135].

## 8. Toxicity Mechanisms

The effects caused by carbon nanoparticles, including GO, can be highly dependent on the organisms and biomarkers considered. The mechanism of toxicity of GO is explained as follows:

### 8.1. Interactions of GO with Cell Membranes

Studies have shown that the interaction between GO and cell membranes is one of the main causes of GO cytotoxicity [140]. Direct contact of the GO with the cells damaged the outer membrane of *E. coli* bacteria and caused the release of intracellular components, leading to cell death [141]. Another study showed that the cytotoxicity of GO is due to direct interactions between the cell membrane and the GO nanosheets that result in physical damage to the cell membrane. Besides, incubation of GO with bovine fetal serum (FBS) reduced the observed damage because of the extremely high protein adsorption capacity of GO [142]. In addition, it has been shown that the interaction of the GO with the lipid membrane is the mechanism for the destructive extraction of membrane lipids. Once GO penetrates the cell, it can destroy high amounts of lipid membrane phospholipids and inducing cell membrane degradation [143].

### 8.2. Oxidative Stress

The toxicity of GO nanosheets is often manifested by the production of reactive oxygen species (ROS), leading to oxidative stress characterized by an imbalance between free radicals and antioxidants. ROSs act as secondary messengers in many intracellular signaling cascades and lead to cellular macromolecular damage, such as degradation of membrane lipids, DNA fragmentation, protein denaturation, and mitochondrial dysfunctions [144]. In a study by Hu and his colleagues, incubating *Euglena gracilis* with GO for ten days caused growth inhibition, decreased photosynthetic pigments, and increased ROS levels [145]. Therefore, the cytotoxic effect of GO on human lung fibroblast (HLF) cells could be due to the oxidative stress that caused apoptosis and DNA damage after exposure of these cells to GO [85]. The accumulation of GO can cause an obstacle to ion channels, leading to the production of ROS. Besides, the treatment of HL-7702 cells with GO resulted in damage to the cell membrane, dependent on the dose and LDH release [146].

**Figure 13:**
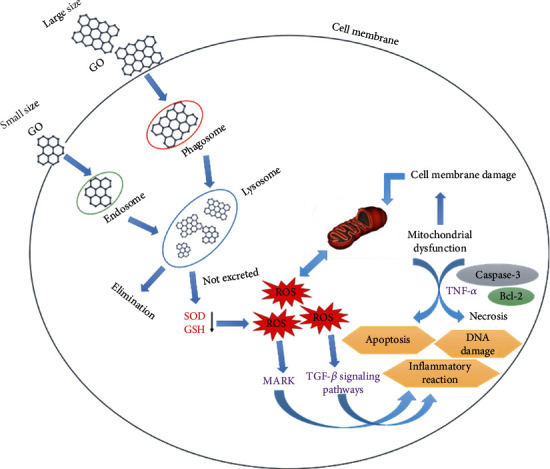
Toxicity mechanisms of GO.

On the other hand, small GO could be degraded by lysosomes and eliminated from the body without causing observable toxicity. On the reverse, large GOs could cause damage to the cell membrane by binding to proteins and interacting with phosphatidylcholine, leading to ROS production, and increasing the dose and duration of exposure to GO results in a progressive decrease in the activity of SOD and GSH (Figure 13). These observed effects can induce a reduction in the ability to eliminate ROS. The generation of ROS in cells treated with GO is the main factor in activating MAPK and the TGF-beta signaling pathways. This activation of these signaling pathways leads to Bim and Bax's activation, which are two proapoptotic members of the Bcl-2 protein family. As a result, caspase-3 and its downstream effector proteins such as PARP were activated, causing mitochondrial dysfunction, DNA damage, inflammatory reactions, apoptosis, and necrosis [147].

## 9. Conclusion

In this review, we have given a detailed overview of the synthesis methods of GO, its structure, different characterization techniques, and its physicochemical properties. Through characterization techniques such as SEM, TEM, and XRD, it has been demonstrated that GO has a nanoscale size. Due to its small size and physicochemical properties, GO is used in several applications, especially biomedical ones. Although GO is useful for many applications, there is still a risk related to its “toxicity,” limiting its uses. Studies conducted so far indicate that the toxicity of GO could depend on its size, synthesis methods, route of administration, and exposure time. In addition, we presented the different toxic effects of this nanomaterial at the cellular and systemic level of the body with discussions on the underlying toxicological mechanism. We also highlighted the role of biological barriers to the entry of GO into the body and its toxicokinetics. ROS-mediated cellular damage has been postulated as a primary mechanism of GO cytotoxicity. In general, available GO toxicity studies are mainly limited to evaluating acute toxicity, while chronic toxicological studies lack. However, the routes of administration, the dose to be administered, and the physicochemical properties directly influence the toxicity of GO. The analysis of these factors allows determining its toxicity. To better understand the toxicological mechanism of this nanoparticle, it is necessary to identify the molecular targets involved in the toxicity and evaluate the benefits and risks of GO for health to benefit from the advantages of nanotechnologies to minimize the risks for human health.

## Figures and Tables

**Table 1:** In vitro cytotoxicity of GO.

Dose of GO (*μ*g/mL)	Cell line	Diameter (nm)	Time (h)	Toxic effect	Reference
3.125-200	Human erythrocytes Human skin fibroblasts CRL-2522	342-765	24	Hemolytic activity, ROS generation, LDH release, decreased cell viability	[86]
5-100	Human fibroblast cells	1 (height)	24	Dose-dependent cytotoxicity, apoptosis	[79]
50–100	Mouse CT26 colon carcinoma cell	Thickness: <2 Lateral size: 450	18	Triggered autophagy, enhances cell death	[96]
100-500	MDA-MB-231	156.4	48	Dose-dependent cytotoxicity; DNA damage, cell cycle arrest, apoptosis	[77]
0–80	HeLa cells	Size distribution: 592 ± 10.9 in PBS, 1272 ± 56.2 in FBS	24	Released LDH, increased MDA and ROS generation, decreased SOD, reduction of cell viability	[97]
20	Macrophage cell J774A.1 THP-1 cells HEK293 cells MEL cells HUT102 cells	Smaller-sized GO: 50-350 Intermediate-sized GO: 350-750 Larger-sized GO: 750-1300	1-24	Size-dependent M1 induction of macrophages, proinflammatory responses	[98]
10-200	Human lung epithelial A549 cells	Thickness of 0.9 Lateral size: s-GO, 160 ± 90 m-GO, 430 ± 300 l-GO, 780 ± 410	24	Dose-dependent oxidative stress, cell viability decreased at high concentration	[80]
7.8, 15.6, 31.2, 62.5, and 125	MCF-7, HUVEC, KMBC/71 cells	100	4-24 h	Significant alterations in the expression level of miR-21, miR-29a, Bax, Bcl2, and PTEN genes after treatment in all three cells Alteration in mitochondrial activity at cellular level	[99]
50	Embryonic stem cell- (ESC-) derived cells	Thickness 1.3	24 h	No significant difference between the level of apoptosis of GO-treated hRPE cells and untreated hRPE controls	[100]

**Table 2:** In vivo toxicity of GO.

Dose of GO	Animals	Diameter (nm)	Time incubation	Toxic effect	Reference
1.0 mg/kg	Male ICR mice	Thickness of 0.9 Size of l-GO: 1-5 *μ*m Size of s-GO: 100-500	Intravenous injected, 24 h	Accumulated mainly in the liver and lungs	[136]
24 mg/kg	Male and female ICR-strain mice	Thickness of <4 Size of l-GO: 237.9 ± 79.3 Size of s-GO: 54.9 ± 23.1	Tail vein injected, 5 days	No effect on the number of pups, sex ratio, weight, survival or growth of pups, and low male reproductive toxicity	[109]
Series concentrations	C57BL/6 male mice	Thickness of 3.9 and 4.05 nm, size of 350 nm and 2 *μ*m	Subcutaneous injection 21 days	The microsize of the GO induced much stronger inflammatory responses than the nanosize of the GO	[84]
0.5 or 4 mg/m^3^	Sprague-Dawley rats	Thickness of 0.93 nm Size of 150–250 nm	Inhalation exposure, single 6	The single inhalation exposure to GO induce minimal toxic responses in rat lungs	[118]
0, 1, 5, 10 mg/kg	C57BL/6 mice	—	Intratracheal instillation 0 h, 24 h, 48 h, 72 h, and 1 week	Leads to acute lung injury and chronic pulmonary fibrosis	[119]
4 mg/kg	Balb/c mice	Thickness of 0.94, 1.22, 4.43, and 5.66; size of 450, 25, 50, and 27	Intraperitoneal injection 1, 7 and 30 days	Accumulated in the reticuloendothelial (RES) system including the liver and spleen over a long time	[103]
5, 10, 20, and 30 g kg^−1^	Earthworms (*Eisenia fetida*)	Thickness of GO 2.1 nm	For 7, 14, 21, and 28 days	Oxidative stress and genotoxicity, resulting in lipid peroxidation, decreased lysosomal membrane stability, and DNA damage	[137]
5, 10, 50, and 100 mg/kg	Male Sprague-Dawley rats	—	Injection into the tail vein once a day for 7 consecutive days	Lung injury in a dose-dependent manner by inducing autophagy	[138]
10, 50, and 100 mg/L	Zebrafish embryos	Diameter 50-200 nm	The embryos were exposed from 6 hpf to 144 hpf in 6-well plates (20 embryos per well)	Neurodevelopmental abnormalities and altered tendency of locomotor in larval fish Increase of AchE and ATPase activities and oxidative stress upregulation and disrupted the expression of genes involved in neurodevelopment and neurotransmitter pathway	[139]
